# Human impact and financial loss of floods in Southeast Asia, from 2007 to 2011

**DOI:** 10.1186/1471-2458-12-S2-A9

**Published:** 2012-11-27

**Authors:** Isidore Koffi Kouadio, Hasanain Faisal Ghazi, Syed Mohamed Aljunid

**Affiliations:** 1 United Nations University-International Institute for Global Health, Universiti Kebangsaan Malaysia Medical Centre, Jalan Yaacob Latiff, 56000 Kuala Lumpur, Malaysia; 2 Department of Community Health, Universiti Kebangsaan Malaysia Medical Centre, Jalan Yaacob Latiff, 56000 Kuala Lumpur, Malaysia

## Background

Flood is the most common (40%) natural disaster worldwide leading occasionally to devastating impact on human and properties. In recent decades, the incidence and magnitude of floods has grown in Southeast Asia region, resulting in substantial economic damages, affecting and killing thousands of people. The aim of this study is to assess the impact of flood on human and estimate the financial loss in Southeast Asia region during the past 5 years.

## Materials and methods

A retrospective cross sectional study was carried out using secondary data from the Centre for Research on the Epidemiology of Disasters (CRED) database (http://www.em-dat.be/). We focused on floods affecting South East Asia’s population from 2007 to 2011.

## Results

A total of 122 floods occurred in 9 countries, with 55% in low income countries and 5% in upper middle income countries. Most of the Floods had occurred in the Philippines (38; 31%) and Indonesia (35; 28.7%). A total of 3016 people were killed (n=90; min=1; max=291) with the highest mortality recorded in October 2010 in Indonesia. A total of 25 873 228 persons were affected by floods (n=114; min= 100; max=8, 970, 653); the highest number resulted from the impact of the 10^th^ October 2010 Thailand’s flood. The regional economic loss from assessment of damages attributed to floods in 6 countries in the region was estimated to 4, 671, 000, 000 USD (n= 42 floods in 6 countries). There was a significant association between economic loss and country’s level of income (p=0.011; 95% CI; 34.47; 253, 54).

## Conclusions

Flood has caused important damages, loss of live and property in Southeast Asia in the past 5 years. The findings of this study suggest to strengthening regional efforts towards flood prevention and mitigation.

